# Disproportionality Analysis of Tirzepatide vs. Semaglutide and Liraglutide: System Organ Class-Level Post-Marketing Reporting Patterns in EudraVigilance

**DOI:** 10.3390/ijms27072988

**Published:** 2026-03-25

**Authors:** Ruxandra Cristina Marin, Cosmin Mihai Vesa, Delia Mirela Tit, Andrei-Flavius Radu, Gabriela S. Bungau

**Affiliations:** 1 Doctoral School of Biological and Biomedical Sciences, University of Oradea, 410087 Oradea, Romania; marin.ruxandracristina@student.uoradea.ro (R.C.M.); andreiflavius.radu@uoradea.ro (A.-F.R.); gbungau@uoradea.ro (G.S.B.); 2 Department of Pharmacology, Clinical Pharmacology and Pharmacotherapy, Faculty of Medicine, “Carol Davila” University of Medicine and Pharmacy, 050474 Bucharest, Romania; 3 Department of Preclinical Disciplines, Faculty of Medicine and Pharmacy, University of Oradea, 410073 Oradea, Romania; 4 Department of Pharmacy, Faculty of Medicine and Pharmacy, University of Oradea, 410028 Oradea, Romania; 5 Department of Psycho-Neuroscience and Recovery, Faculty of Medicine and Pharmacy, University of Oradea, 410073 Oradea, Romania

**Keywords:** tirzepatide, semaglutide, liraglutide, dual GIP/GLP-1 receptor agonist, incretin signaling, post-marketing safety, pharmacovigilance, EudraVigilance, reporting odds ratio

## Abstract

Tirzepatide, a dual glucose-dependent insulinotropic polypeptide (GIP) and glucagon-like peptide 1 (GLP-1) receptor agonist, introduces a mechanistically distinct approach within incretin-based therapies. While its efficacy is established, real-world data comparing post-marketing safety with established GLP-1 receptor agonists remain limited. This study assessed System Organ Class (SOC)-level reporting patterns for tirzepatide versus semaglutide and liraglutide using EudraVigilance data. Aggregated individual case safety reports (ICSRs) were analyzed using pairwise disproportionality analyses based on a case/non-case approach. Reporting odds ratios (RORs) with 95% confidence intervals were calculated. False discovery rate (FDR) correction using the Benjamini–Hochberg procedure and sensitivity analyses restricted to serious and healthcare professional–reported cases were performed to assess robustness. After FDR adjustment, 20 SOCs were significant in tirzepatide–semaglutide and 23 in tirzepatide–liraglutide comparisons; eight SOCs remained significant across all analytical conditions. Compared with semaglutide, tirzepatide showed higher reporting for immune (ROR 1.97, 95% CI 1.75–2.21) and hepatobiliary disorders (ROR 1.71, 95% CI 1.61–1.82). Versus liraglutide, higher odds occurred for musculoskeletal (ROR 2.02, 95% CI 1.85–2.21) and psychiatric disorders (ROR 2.14, 95% CI 1.99–2.30), and lower odds for neoplasms (ROR 0.28, 95% CI 0.26–0.31). Tirzepatide shows heterogeneous reporting patterns compared with GLP-1 receptor agonists, with consistent excess reporting for hepatobiliary, immune, and musculoskeletal disorders. These findings are hypothesis-generating and warrant confirmation in exposure-adjusted studies.

## 1. Introduction

Incretin hormones, notably glucagon-like peptide-1 (GLP-1) and glucose-dependent insulinotropic polypeptide (GIP), are central regulators of postprandial glucose homeostasis, appetite suppression, and energy balance, acting through G protein–coupled receptor signaling pathways that modulate pancreatic insulin secretion, glucagon suppression, and gastrointestinal motility. Beyond these classical metabolic roles, incretin receptors are widely expressed in extra-pancreatic tissues, including the central nervous system, cardiovascular system, adipose tissue, and immune cells, suggesting that pharmacological incretin receptor modulation may exert systemic effects across multiple organ systems [1].

Glucagon-like peptide-1 receptor agonists (GLP-1 RAs), such as semaglutide and liraglutide, have been successfully integrated into clinical practice for the management of type 2 diabetes mellitus and obesity, demonstrating consistent efficacy in glycemic control and weight reduction, alongside beneficial effects on cardiovascular risk factors [2].

Beyond their metabolic efficacy, GLP-1 RAs have also demonstrated anti-inflammatory and potential neuroprotective effects in preclinical and clinical studies, further supporting the concept that GLP-1 receptor signaling mediates biologically diverse actions beyond glucose regulation alone [3].

Despite their established clinical utility, the full safety profiles of GLP-1 RAs remain incompletely characterized, particularly with respect to rare, delayed, or organ-specific adverse events that may not be detected within the controlled setting of randomized clinical trials but emerge during widespread real-world use. This limitation is well recognized in pharmacovigilance research and highlights the importance of complementary real-world evidence [4].

Tirzepatide represents a pharmacologically distinct advancement within incretin-based therapies as the first approved dual agonist of both GIP and GLP-1 receptors. Developed as a single unimolecular peptide targeting both incretin pathways, tirzepatide has demonstrated superior glycemic reduction and weight loss compared with selective GLP-1 receptor agonists in phase III clinical trials, including the SURPASS and SURMOUNT programs [4,5].

Given its dual receptor activity, tirzepatide raises important questions regarding how simultaneous GIP and GLP-1 receptor engagement may influence biological processes beyond glycemic control, including pathways related to inflammation, adipose tissue biology, and cardiovascular modulation [1].

**Figure 1:**
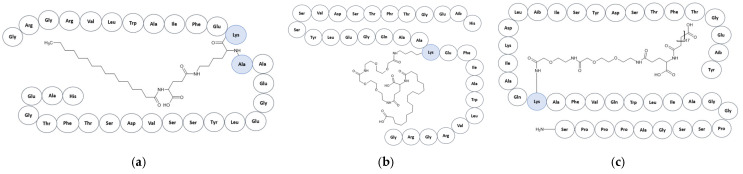
Chemical structures of incretin-based peptide therapeutics. (**a**), liraglutide; (**b**), semaglutide; (**c**), tirzepatide. Structures were generated using ACD/ChemSketch (version 12.1.0.31258), Advanced Chemistry Development, Inc., Toronto, ON, Canada. The figures illustrate the peptide backbone and key structural modifications, including amino-acid substitutions and lipid side-chain conjugations responsible for prolonged half-life and receptor activity.

Although liraglutide, semaglutide, and tirzepatide belong to the incretin-based therapeutic class, their molecular architectures differ and contribute to variations in pharmacokinetics and receptor pharmacology (Figure 1a–c). Liraglutide is a long-acting GLP-1 analogue containing a C16 fatty-acid side chain that promotes albumin binding and prolonged circulation. Semaglutide incorporates additional amino-acid substitutions (e.g., α-aminoisobutyric acid (Aib) at position 8) and a longer C18 fatty diacid chain that further increases molecular stability and extends half-life. In contrast, tirzepatide is a 39-amino-acid peptide designed to activate both glucose-dependent insulinotropic polypeptide (GIP) and GLP-1 receptors and contains a C20 fatty diacid moiety that enables extended systemic exposure. These structural differences may influence receptor signaling and pharmacodynamic effects among the agents [6,7,8].

Preclinical studies indicate that co-activation of GIP and GLP-1 receptors may produce functionally selective signaling responses, including differences in cyclic AMP signaling and β-arrestin recruitment that influence receptor internalization and trafficking dynamics [6,9]. However, such molecular mechanisms cannot be directly evaluated using aggregated pharmacovigilance data. Disproportionality analyses based on spontaneous reporting systems are designed for signal detection and hypothesis generation rather than for establishing mechanistic or causal relationships. In this context, biomolecular insights are presented primarily to provide biological background for potential pharmacological differences between dual and selective incretin receptor agonists rather than to imply that receptor-level signaling can be directly inferred from spontaneous reporting patterns [10].

At a systems-biology level, signaling diversity at class B G protein–coupled receptors may contribute to tissue-specific pharmacological responses [9,11]. Nevertheless, pharmacovigilance analyses based on System Organ Class–level reporting patterns cannot resolve receptor-level signaling mechanisms. Instead, such analyses should be interpreted as hypothesis-generating approaches that may reveal differences in real-world safety reporting profiles across related therapies and guide subsequent mechanistic or pharmacoepidemiologic investigations [12].

Against this pharmacologic background, the extent to which such tissue-selective and context-dependent signaling differences translate into discernible multi-organ safety patterns in routine clinical practice remains insufficiently characterized. Although tirzepatide’s clinical profile continues to be refined through post-authorization experience, comparative real-world evidence directly contrasting its broader safety and biological footprint with established GLP-1 receptor agonists remains limited. In particular, few observational studies have systematically examined whether dual incretin receptor activation is associated with distinct multi-organ adverse event patterns compared with single-receptor agonists, and analyses at the System Organ Class level are especially scarce. This gap underscores the need for robust pharmacovigilance studies that integrate real-world data with mechanistic considerations [13].

The clinical use of incretin-based therapies has expanded markedly across Europe in recent years, reflecting their approved roles in type 2 diabetes and chronic weight management as well as broader cardiometabolic prevention strategies. Although comprehensive EU-wide prescription data are not yet consolidated in a single registry, population-based estimates from Great Britain indicate that approximately 4.5% of adults were using GLP-1 or GLP-1/GIP medications by early 2025, corresponding to about 1.6 million individuals, with a substantial proportion using these agents for weight management. This rapid uptake has coincided with persistent shortages of GLP-1 receptor agonists across multiple EU Member States since 2022, prompting coordinated regulatory responses [14,15,16].

National prescribing data further illustrate this trend. In Stockholm, Sweden, the prevalence of GLP-1 receptor agonist dispensations increased from 4.7 per 1000 inhabitants in 2019 to 17.5 per 1000 in 2023, highlighting substantial growth in real-world use within a major European healthcare system [17].

European utilization patterns mirror broader global trends, with recent analyses estimating that approximately 27% of adults worldwide may be eligible for GLP-1-based therapies for weight management, reflecting both the global burden of obesity and the expanding therapeutic indications for incretin-based agents [18].

Incretin-based therapies are also increasingly used as adjunct options after bariatric surgery, particularly in patients experiencing insufficient weight loss or weight regain. Real-world studies suggest that GLP-1 receptor agonists can support additional weight reduction in this setting, and early clinical experience indicates growing interest in dual GIP/GLP-1 receptor agonists for post-surgical weight recurrence [19]. This approach aligns with broader evidence highlighting the role of integrated metabolic and bariatric strategies in improving long-term outcomes and quality of life in patients with obesity [20].

In this context of rapidly expanding and heterogeneous real-world use, systematic evaluation of post-marketing safety data becomes essential to assess whether pharmacologically related incretin-based therapies exhibit distinct patterns of organ-specific adverse event reporting that may reflect differences in underlying receptor engagement and signaling mechanisms. Pharmacovigilance databases such as EudraVigilance provide a framework for real-world signal detection through the analysis of spontaneous adverse event reports, enabling hypothesis generation regarding differential safety profiles across related therapies [10,21].

Previous comparative pharmacovigilance investigations using EudraVigilance data have demonstrated that disproportionality analyses can identify distinct organ-system reporting patterns across therapeutic classes and inform real-world safety evaluation of newly introduced or repurposed agents [22].

Thus, comparative analyses of System Organ Class–level (SOC-level) reporting patterns may help determine whether dual GIP/GLP-1 receptor activation with tirzepatide is associated with differentiated multi-organ safety-reporting profiles compared with established GLP-1 receptor agonists. Evaluating these patterns within a pharmacovigilance framework may provide additional context for interpreting potential biological and pharmacodynamic differences between dual and single incretin receptor agonism.

Accordingly, the aim of the present study was to systematically compare SOC-level post-marketing reporting patterns of tirzepatide with those of semaglutide and liraglutide using aggregated data from the EudraVigilance database, through pairwise disproportionality analyses.

## 2. Results

### 2.1. Demographic Characteristics of Individual Case Safety Reports

A total of 94,866 individual case safety reports (ICSRs) related to the three incretin-based therapies included in the analysis were retrieved from the EudraVigilance database. Of these, 26,483 reports were associated with tirzepatide, 47,337 with semaglutide, and 21,046 with liraglutide.

The distribution of ICSRs by age group is summarized in Table 1. Across all three agents, the majority of reports concerned adult patients aged 18–64 years, accounting for 48.2% of tirzepatide, 43.7% of semaglutide, and 46.6% of liraglutide reports. Reports involving patients aged 65–85 years represented 11.0%, 19.7%, and 16.5% of cases, respectively, while reports in patients older than 85 years were uncommon, accounting for less than 1% of ICSRs for all three drugs. Pediatric reports (<18 years) were rare overall, although they were slightly more frequent for semaglutide and liraglutide than for tirzepatide. A substantial proportion of ICSRs lacked age information, with missing data ranging from 35.6% to 40.3% across the three medicinal products, as detailed in Table 1.

Sex distribution of ICSRs is presented in Table 2. A consistent female predominance was observed for all three agents, with female patients accounting for 63.2% of tirzepatide, 59.1% of semaglutide, and 60.7% of liraglutide reports. Male patients represented approximately one-quarter to one-third of reports, while sex was not specified in 6.9% to 13.3% of cases across the three drugs.

### 2.2. Geographic Origin and Reporter Type

The geographic origin of individual case safety reports (ICSRs) is summarized in Table 3. Across all three incretin-based therapies, the majority of reports originated from countries outside the European Economic Area (non-EEA). Non-EEA reports accounted for 68.6% of tirzepatide, 57.0% of semaglutide, and 59.6% of liraglutide ICSRs, while reports originating from EEA countries represented approximately one-third to two-fifths of cases for each agent.

The distribution of ICSRs according to reporter type is presented in Table 4. Healthcare professionals were the primary reporters for all three agents, particularly for tirzepatide and liraglutide, accounting for 61.3% and 64.1% of reports, respectively. In contrast, semaglutide exhibited a more balanced reporting pattern, with a substantial proportion of reports submitted by non-healthcare professionals, who accounted for 48.5% of ICSRs for this agent.

### 2.3. Overall Seriousness of Reported Cases

The overall seriousness classification of individual case safety reports (ICSRs) is summarized in Table 5. For all three incretin-based therapies, the majority of reports were classified as serious, accounting for 73.5% of tirzepatide, 66.4% of semaglutide, and 73.5% of liraglutide ICSRs. Non-serious reports represented 26.5% of tirzepatide, 33.6% of semaglutide, and 26.5% of liraglutide cases. The corresponding serious-to-non-serious reporting ratios ranged from 1.97 for semaglutide to 2.77 for both tirzepatide and liraglutide.

### 2.4. Distribution of Reports by System Organ Class

The distribution of ICSRs according to SOC is presented in Table 6. Across all three incretin-based therapies, gastrointestinal disorders represented the most frequently reported SOC, accounting for approximately half of all SOC-linked reports. Other commonly reported SOCs included general disorders and administration site conditions, injury, poisoning and procedural complications, metabolism and nutrition disorders, nervous system disorders, and psychiatric disorders.

Although the overall pattern of frequently reported SOCs was broadly similar across tirzepatide, semaglutide, and liraglutide, differences were observed in the relative contribution of specific SOCs among the three agents.

### 2.5. Reporting Complexity

Overall reporting complexity, expressed as the mean number of SOCs reported per ICSR, is summarized in Table 7. The mean number of SOCs per report was 1.82 for tirzepatide, 2.05 for semaglutide, and 1.83 for liraglutide, with an overall mean of 1.94 across all ICSRs included in the analysis.

The observed differences in reporting complexity across the three agents were modest. Together, these descriptive findings provide the necessary context for the interpretation of subsequent disproportionality analyses, which examine differences in reporting odds across System Organ Classes among tirzepatide, semaglutide, and liraglutide.

### 2.6. Results of Disproportionality Analysis

#### 2.6.1. Comparative SOC-Level Disproportionality Profiles of Tirzepatide vs. Semaglutide and Liraglutide

Pairwise disproportionality analyses comparing tirzepatide with semaglutide and liraglutide revealed distinct SOC-level reporting patterns across the EudraVigilance database. In the comparison between tirzepatide and semaglutide, several SOCs demonstrated significantly higher reporting odds for tirzepatide, as indicated by RORs greater than unity with 95% confidence intervals excluding 1.0 (Table 8). In particular, increased reporting odds were observed for immune system disorders, hepatobiliary disorders, musculoskeletal and connective tissue disorders, reproductive system and breast disorders, blood and lymphatic system disorders, skin and subcutaneous tissue disorders, and vascular disorders.

Conversely, lower reporting odds for tirzepatide relative to semaglutide were identified across multiple SOCs, including product issues, surgical and medical procedures, social circumstances, injury, poisoning and procedural complications, eye disorders, metabolism and nutrition disorders, and ear and labyrinth disorders, several of which showed marked reductions in reporting odds (ROR < 0.6). For other SOCs, including cardiac disorders, renal and urinary disorders, and infections and infestations, reporting odds were broadly comparable between the two agents, with confidence intervals overlapping unity.

When compared with liraglutide, tirzepatide exhibited more pronounced and widespread differences in reporting odds across SOCs (Table 8). Significantly increased reporting odds for tirzepatide were observed for numerous SOCs, including reproductive system and breast disorders, psychiatric disorders, musculoskeletal and connective tissue disorders, skin and subcutaneous tissue disorders, vascular disorders, respiratory, thoracic and mediastinal disorders, immune system disorders, and hepatobiliary disorders, with several RORs exceeding 2.

In contrast, tirzepatide showed substantially lower reporting odds relative to liraglutide for selected SOCs, most notably neoplasms that are benign, malignant and unspecified, product issues, surgical and medical procedures, social circumstances, and congenital, familial and genetic disorders, where RORs were markedly below unity.

The magnitude of significant ROR estimates ranged from modest elevations (approximately 1.1–1.5) to more pronounced signals exceeding 2.0 in selected SOCs, indicating variable strength of disproportionality across organ systems. Reporting odds ratios (RORs) with 95% confidence intervals for all SOCs are presented in Table 8.

Across both pairwise analyses, tirzepatide displayed a distinct SOC-level disproportionality profile, characterized by: SOCs with consistently increased reporting odds relative to one or both comparators, SOCs with reduced reporting odds, and SOCs with largely comparable reporting odds across agents.

**Figure 2:**
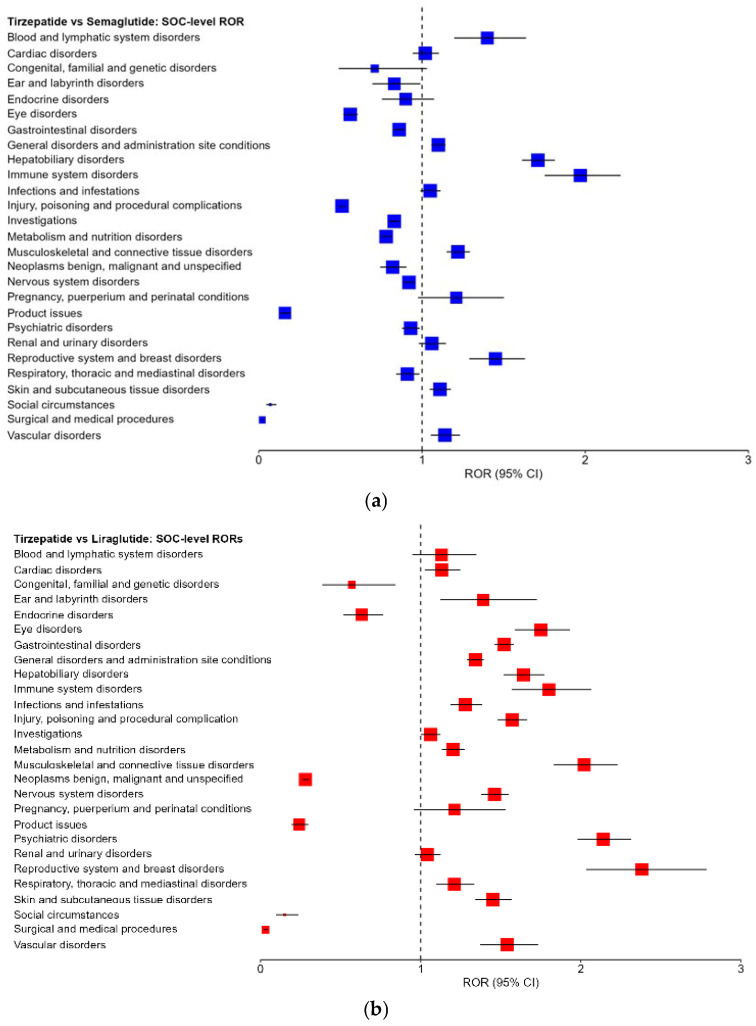
Forest plots of reporting odds ratios (RORs) comparing tirzepatide with semaglutide (**a**) and liraglutide (**b**) across System Organ Classes (SOCs) using EudraVigilance data. Squares represent ROR estimates and horizontal lines indicate 95% confidence intervals. The vertical dashed line corresponds to ROR = 1, indicating no difference in reporting odds.

Overall, visual inspection of the forest plots (Figure 2a,b) highlights that both the number and magnitude of significant disproportionality signals were greater in the comparison between tirzepatide and liraglutide than in the comparison between tirzepatide and semaglutide, indicating differential post-marketing reporting patterns among these incretin-based therapies.

#### 2.6.2. Robustness and Multiplicity-Adjusted Analysis

To account for multiple testing across SOCs, false discovery rate (FDR) correction using the Benjamini–Hochberg procedure was applied separately within each pairwise comparison. After FDR adjustment, 20 SOC-level signals remained statistically significant in the tirzepatide versus semaglutide comparison and 23 in the tirzepatide versus liraglutide comparison.

Sensitivity analyses restricted to serious reports demonstrated persistence of 18 of 20 signals (90%) in the tirzepatide versus semaglutide comparison. In the tirzepatide versus liraglutide comparison, 18 of 23 signals (78.3%) remained statistically significant after restriction to serious reports. When further restricted to reports submitted by healthcare professionals, 15 SOCs remained fully robust in the tirzepatide versus semaglutide comparison and 11 SOCs in the tirzepatide versus liraglutide comparison.

Overall, stratified analyses restricted to serious reports and to healthcare professional–submitted reports showed consistent directionality of SOC-level signals, with moderate attenuation or amplification of effect sizes in selected organ systems (Supplementary Table S1).

Across both pairwise comparisons, eight SOCs remained fully robust under all analytical conditions (FDR-adjusted primary analysis, serious-only restriction, and healthcare professional–restricted analysis). These included eye disorders, gastrointestinal disorders, hepatobiliary disorders, immune system disorders, injury, poisoning and procedural complications, metabolism and nutrition disorders, musculoskeletal and connective tissue disorders, and neoplasms that are benign, malignant and unspecified.

Among these, hepatobiliary disorders, immune system disorders, and musculoskeletal and connective tissue disorders demonstrated consistently elevated reporting odds ratios in both comparisons, representing the most stable excess disproportionality signals associated with tirzepatide. In contrast, neoplasms demonstrated consistently reduced reporting odds ratios relative to both comparators, while the remaining SOCs exhibited comparator-dependent directionality.

Table 9 presents the intersection of fully robust SOC-level signals, defined as those remaining statistically significant after FDR correction and persisting under both serious-case and healthcare professional–restricted analyses in both pairwise comparisons.

**Figure 3:**
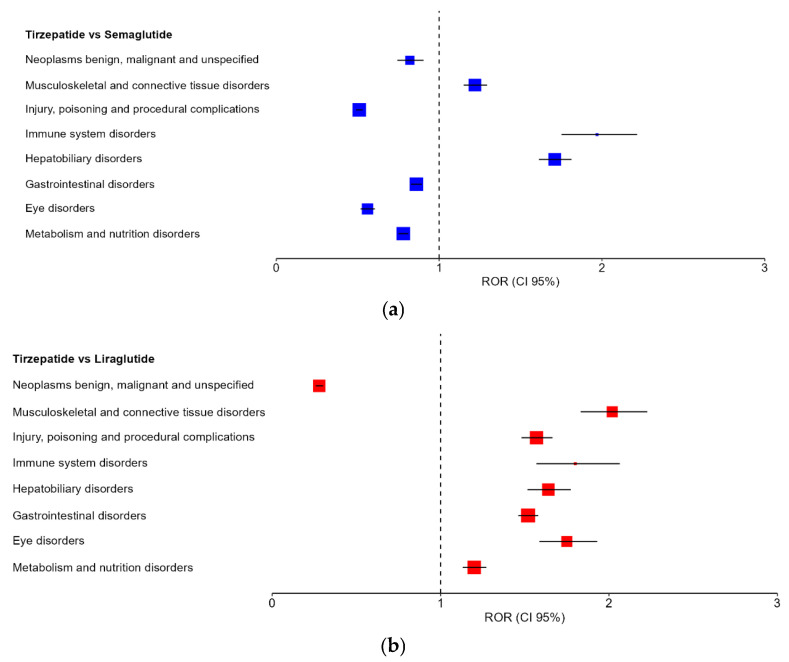
Forest plots of fully robust SOC-level disproportionality signals. (**a**), tirzepatide vs. semaglutide; (**b**), tirzepatide vs. liraglutide. Squares represent reporting odds ratios (RORs) and horizontal lines indicate 95% confidence intervals. The vertical dashed line corresponds to ROR = 1, indicating no difference in reporting odds.

To further illustrate the stability and directionality of these high-confidence signals, forest plots restricted to the eight fully robust SOCs are presented in Figure 3. These plots visually demonstrate the consistency of effect direction across comparisons, highlighting SOCs with persistent excess reporting (ROR > 1 in both comparisons), those with consistently reduced reporting (ROR < 1), and SOCs exhibiting comparator-dependent directionality.

Notably, the comparison between tirzepatide and liraglutide yielded a greater number and magnitude of significant disproportionality signals than the comparison with semaglutide.

To improve the clinical interpretability of SOC-level findings, we examined the most frequently reported preferred terms (PTs) within the eight fully robust SOCs using EudraVigilance line-listing data. Across these SOCs, several PTs corresponded to clinically recognized effects associated with incretin-based therapies. For example, hepatobiliary reports were primarily driven by gallbladder-related events such as cholelithiasis and cholecystitis. Gastrointestinal disorders were dominated by abdominal pain, diarrhoea, nausea, and pancreatitis-related PTs across all three agents. Immune system reports mainly reflected hypersensitivity and anaphylactic reactions, while musculoskeletal disorders were largely represented by arthralgia and back pain. Within metabolism and nutrition disorders, dehydration, decreased appetite, and hypoglycaemia were the most frequent PTs. A descriptive overview of the leading PTs within each SOC is presented in Table 10.

### 2.7. Cross-Source Evaluation of SOC-Level Adverse Event Signals

To contextualize the disproportionality findings, SOC-level signals identified in the EudraVigilance database were systematically examined against regulatory labeling information (Summary of Product Characteristics, SmPC) and published clinical evidence. This triangulation approach was undertaken to assess the concordance between pharmacovigilance signals, established safety profiles, and findings from randomized trials and observational studies. The comparative synthesis across spontaneous reporting data, SmPC information, and literature evidence is summarized in Table 11.

## 3. Discussion

In this large-scale pharmacovigilance analysis based on EudraVigilance data, we identified consistent and biologically plausible differences in SOC-level reporting patterns for tirzepatide compared with the established GLP-1 receptor agonists semaglutide and liraglutide. Although disproportionality analyses do not establish causality, the persistence of multiple signals across primary, FDR-adjusted, serious-case, and healthcare professional–restricted analyses supports the robustness of the observed reporting heterogeneity. These findings suggest that post-marketing safety profiles within the incretin class are not uniform and may reflect both pharmacologic and contextual determinants. Importantly, the interpretation of SOC-level signals in spontaneous reporting systems should remain anchored in their hypothesis-generating purpose, given the absence of exposure denominators and the susceptibility to stimulated reporting and reporting-quality heterogeneity across regions and reporter groups [12,23]. This interpretative framework is consistent with current pharmacovigilance methodological recommendations, including the READUS-PV guidance, which emphasize transparency, robustness analyses, and the hypothesis-generating nature of disproportionality findings [10].

In this context, the unique dual GIP/GLP-1 receptor agonist mechanism of tirzepatide provides a biologically plausible framework for interpreting the observed multi-organ reporting profiles and for generating hypotheses that warrant further investigation in complementary pharmacoepidemiologic and mechanistic studies. This biomolecular framing is particularly relevant when comparative disproportionality suggests organ-system “signatures” that persist across multiple robustness restrictions, as observed here [6,24].

The demographic and reporting characteristics provide important context for interpreting disproportionality findings. An additional factor influencing spontaneous reporting patterns is stimulated reporting during the early post-marketing period, commonly referred to as the Weber effect. Newly approved medicines often experience increased adverse-event reporting shortly after market introduction due to heightened regulatory scrutiny, clinician awareness, and pharmacovigilance monitoring, which may inflate reporting frequencies independently of true pharmacological risk [10,25].

Because tirzepatide received European marketing authorization more recently than semaglutide and liraglutide, differences in time-on-market and cumulative patient exposure may partially influence comparative reporting patterns observed in pharmacovigilance databases. Across all three therapies, ICSRs predominantly involved adults, with fewer reports in elderly and pediatric populations, consistent with prescribing patterns and the known underrepresentation of certain age groups in spontaneous reporting systems [24,26].

The observed female predominance may reflect higher healthcare utilization, biological differences, and reporting behavior among women. Sex-related disparities in adverse drug reaction reporting are well documented across therapeutic classes, so sex distribution should be interpreted as reflecting reporting propensity rather than sex-specific causal susceptibility at the SOC level [27].

Differences in geographic origin and reporter type highlight reporting heterogeneity. The higher proportion of non-EEA reports and the substantial contribution of patient-reported ICSRs, particularly for semaglutide, may influence both the nature and frequency of reported events. As demographic composition, regional practices, and reporter type can shape reporting patterns, disproportionality signals should be interpreted within the broader clinical and surveillance context rather than as direct indicators of drug-specific risk [26,28].

Importantly, eight SOCs remained fully robust across all analytical conditions. Among these, hepatobiliary disorders, immune system disorders, and musculoskeletal and connective tissue disorders demonstrated consistently elevated reporting odds for tirzepatide relative to both comparators, representing the most stable excess disproportionality signals. Conversely, neoplasms showed consistently reduced reporting odds across comparisons. The identification of such cross-validated SOC-level signals strengthens the interpretability of the findings and reduces the likelihood that they are driven solely by reporting noise or single-stratum artifacts. Linking these robust SOCs back to the descriptive results, the overall predominance of serious reports across agents and the modest differences in reporting complexity (multiple SOCs per report) suggest that signal persistence is unlikely to be explained solely by differential “case richness,” reinforcing the value of the multi-restriction robustness approach. The PT-level descriptive analysis further contextualized these SOC-level signals, indicating that several robust SOC patterns were driven by clinically recognizable event clusters, such as gallbladder-related events within hepatobiliary disorders, hypersensitivity reactions within immune system disorders, and musculoskeletal pain–related terms within musculoskeletal disorders.

The discrepancy between the total number of FDR-confirmed signals in the primary analysis and those retained in the serious-only sensitivity analysis reflects the impact of case-mix restriction on disproportionality estimates. Restricting the dataset to serious reports reduces the number of cases and alters the distribution of events across SOCs, leading to reduced statistical power and attenuation of certain signals. Consequently, some associations that met the FDR-adjusted significance threshold in the full dataset no longer satisfied the criteria under the serious-only restriction, despite maintaining similar effect directions. In addition, differences in reporting patterns between serious and non-serious cases, including differential reporting probabilities and clinical prioritization of severe events, may further contribute to the observed differences [12,24]. These pharmacovigilance-derived system-level signals warrant cautious biological consideration. Tirzepatide’s dual GIP/GLP-1 receptor agonist pharmacology represents a potential contextual framework for interpreting differences in reporting patterns. However, the present pharmacovigilance design does not permit direct inference about underlying biological mechanisms. The observed SOC-level signals may reflect a combination of factors, including pharmacological differences, variation in treated patient populations or indications, time-on-market asymmetry, and differences in surveillance or reporting dynamics. Consequently, such interpretations should be regarded as hypothesis-generating rather than explanatory. The directional stability of signals across serious-only and healthcare professional–restricted analyses supports the robustness of the disproportionality findings but does not establish a causal biological explanation [9,29].

Gastrointestinal disorders represented the most frequently reported SOC across all agents, consistent with the well-established class effects of incretin-based therapies. However, the divergent reporting patterns, lower odds for tirzepatide versus semaglutide but higher versus liraglutide, suggest that gastrointestinal tolerability within the incretin class is not homogeneous, and may vary according to pharmacodynamic potency, receptor signaling profiles, and dose-escalation strategies [4]. Differences in dose-escalation schedules among these agents may also contribute to variation in GI adverse-event reporting, as slower titration strategies are commonly used to mitigate nausea and other gastrointestinal symptoms during treatment initiation [30].

Experimental data indicate that GIP receptor activation may modulate GLP-1–mediated gastrointestinal responses via central vagal and enteric mechanisms, potentially attenuating nausea while preserving metabolic efficacy. Such receptor cross-talk may contribute to qualitative differences in gastrointestinal adverse-event reporting [6,9].

The most consistent excess signal for tirzepatide involved hepatobiliary disorders. Gallbladder and biliary events have been associated with GLP-1 receptor agonists in randomized evidence syntheses, particularly in weight-loss contexts. Recent systematic evidence supports a dose-, duration-, and indication-dependent association between GLP-1 receptor agonist exposure and gallbladder/biliary outcomes, strengthening the plausibility that differential weight-loss trajectories across agents may translate into differential reporting patterns [31]. It should also be considered that differences in treatment indication (type 2 diabetes versus obesity management) may contribute to variation in baseline patient characteristics, including body mass index and comorbidity burden, which could influence reporting patterns for some System Organ Classes.

The magnitude and rapidity of weight reduction achieved with dual incretin receptor agonism may amplify gallstone formation risk through alterations in bile composition and gallbladder motility [5].

While GLP-1 signaling is known to influence hepatic lipid metabolism and bile acid pathways, the independent role of GIP receptor activation in biliary physiology remains unclear. The observed signal is therefore biologically plausible but likely reflects interactions between pharmacology and rapid metabolic change rather than direct hepatotoxicity. Meta-analyses of tirzepatide also suggest a possible increase in gallbladder or biliary events, though estimates vary. Future exposure-adjusted studies should distinguish receptor-specific effects, weight-loss–related changes, and baseline gallstone risk factors to clarify causality [32,33].

At the molecular level, GLP-1 receptor signaling has been implicated in the regulation of hepatic lipid metabolism, bile acid synthesis, and gallbladder motility through pathways involving AMPK activation and FXR–FGF19 signaling, whereas the independent contribution of GIP receptor activation to biliary physiology remains insufficiently characterized. This mechanistic uncertainty underscores why the hepatobiliary SOC signal should be treated as a clinically relevant monitoring hypothesis rather than definitive evidence of a direct drug-induced hepatic/biliary toxicity pathway [34].

Signals within metabolism and nutrition and endocrine SOCs require cautious interpretation, as these categories capture both expected pharmacodynamic effects and surveillance-driven reporting. Lower reporting odds for metabolism-related events versus semaglutide but higher versus liraglutide underscore that metabolic reporting heterogeneity is comparator-dependent [35].

Hypoglycemia risk remains largely context-dependent and aligns with the glucose-dependent insulinotropic action of tirzepatide, supporting the interpretation that many metabolic signals reflect treatment context rather than intrinsic toxicity. This also highlights the limitations of SOC-level aggregation for outcomes that are heavily modified by concomitant insulin or sulfonylurea exposure [30].

Lower reporting odds for endocrine disorders with tirzepatide versus liraglutide may reflect differences in event composition—particularly thyroid-related reports—and variations in surveillance. Evidence linking GLP-1 receptor agonists to thyroid cancer is mixed, so differential reporting likely reflects monitoring intensity rather than drug-specific endocrine toxicity. Accordingly, reduced neoplasm reporting with tirzepatide is more consistent with legacy regulatory surveillance and historical safety monitoring of earlier GLP-1 receptor agonists than with evidence of comparative cancer protection [36,37]. This observation illustrates how SOC-level signals in spontaneous reporting systems may be shaped by regulatory attention and reporting context rather than reflecting intrinsic pharmacological differences between agents.

Similarly, the “Investigations” SOC often reflects monitoring intensity rather than direct pharmacologic toxicity, particularly during rapid weight loss, dose escalation, or comorbidity follow-up. This is especially relevant when reports include laboratory abnormalities without standardized timing, baseline values, or exposure duration, limiting causal interpretation [38].

Neuropsychiatric and nervous system signals were comparator-dependent and should be interpreted carefully. Central incretin signaling influences appetite, reward processing, and autonomic regulation. Emerging neurobiological data suggest that dual incretin receptor activation may modulate hypothalamic and mesolimbic circuits differently from selective GLP-1 receptor agonism [39].

Nevertheless, psychiatric reporting is particularly sensitive to indication mix, baseline comorbidity, and regulatory attention. Current regulatory assessments have not established a causal link with suicidality, emphasizing the need for outcome-specific analyses rather than SOC-level inference alone [40].

Comparable pharmacovigilance studies show that spontaneous reporting systems can detect heterogeneous neuropsychiatric event clusters but remain susceptible to confounding from disease severity, psychiatric comorbidity, and reporting awareness. Regulatory assessments have not established a causal link with suicidality, and publicity effects may temporarily influence reporting, supporting cautious interpretation of psychiatric SOC signals [41,42,43].

Cardiac and renal reporting patterns were broadly similar for tirzepatide and semaglutide, aligning with emerging outcome data suggesting cardiometabolic benefit rather than excess harm. Robust randomized evidence, including the SELECT trial, supports cardiovascular benefit for semaglutide and provides a benchmark for interpreting SOC-level cardiac reporting. These largely neutral patterns also reflect the limited sensitivity of spontaneous reporting systems for detecting benefit, highlighting the complementary role of outcomes trials and exposure-adjusted observational studies [44].

Exploratory findings from SURPASS-4 also suggest potential kidney-protective effects, including slower eGFR decline and reduced albuminuria, which may not be fully captured at the SOC level. Thus, the absence of excess cardiac or renal reporting should be interpreted as consistent with current evidence rather than proof of equivalence [45,46].

The vascular SOC signal observed with tirzepatide is difficult to interpret due to event heterogeneity and confounding by rapid weight loss, dehydration, antihypertensive adjustments, and baseline cardiometabolic risk. Mechanistic and trial evidence overall supports vascular risk improvement with incretin therapies, highlighting the limitations of SOC-level aggregation for cardiovascular inference [47].

Tirzepatide showed higher reporting odds for immune and respiratory disorders than liraglutide. Immune-related reports likely reflect injection-site or mild hypersensitivity reactions, which are known but infrequent with GLP-1–based therapies. Although anti-drug antibodies may occur, they are rarely associated with loss of efficacy or severe hypersensitivity, suggesting these signals primarily reflect reporting of mild-to-moderate reactions rather than serious systemic immune toxicity [48,49].

Respiratory SOC disproportionality likely reflects heterogeneous event composition and clinical context rather than direct drug effects. Randomized and observational evidence has not demonstrated an increased overall infection risk with GLP-1 receptor agonists and, in some settings, suggests neutral or protective associations for specific respiratory outcomes [50,51].

Markedly lower reporting odds for pregnancy-related and congenital SOCs with tirzepatide are best explained by restricted use and limited exposure rather than intrinsic fetal safety. Although inadvertent early pregnancy exposure may occur, recent observational data for GLP-1 receptor agonists have not indicated an increased risk of major congenital malformations, though agent-specific data for dual agonists remain limited [52,53,54].

The triangulated evaluation of pharmacovigilance signals against regulatory labeling (SmPC) and contemporary literature provides an integrated basis for interpreting disproportionality patterns. Concordance for key SOCs, particularly hepatobiliary, gastrointestinal, and immune-related events, suggests many reports align with recognized incretin-related physiological effects and established monitoring practices, especially in contexts of substantial weight reduction and metabolic change. Post-2020 evidence further indicates that biliary events, gastrointestinal intolerance, and injection-site/immunogenic reactions are strongly shaped by treatment context, dose, and duration rather than uniform drug-specific toxicity, with meta-analyses supporting gallbladder-related risk in weight-loss settings and phase 3 data indicating generally limited clinical consequences of treatment-emergent immunogenicity [31,49].

At the same time, divergences between spontaneous reports and labeling likely reflect differential exposure, surveillance intensity, reporting awareness, and evolving prescribing patterns; thus, signals should be interpreted alongside regulatory attention and underlying disease risk. Population-based analyses also suggest that some historically monitored outcomes (e.g., thyroid-related) have not consistently translated into increased clinical incidence, underscoring the need to separate surveillance-driven reporting from true harm. Within this framework, persistent cross-stratum SOC differences may indicate a distinct real-world safety-reporting signature for dual GIP/GLP-1 receptor agonism, but confirmation requires exposure-adjusted studies, time-to-event analyses, and mechanistic research [24,31,49,55,56].

Several limitations should be considered. Spontaneous reporting systems are inherently subject to reporting bias, including stimulated reporting and temporal changes in safety awareness, which may influence signal detection independently of true clinical risk. Differences in time on market, therapeutic indications, prescribing practices, and patient characteristics across the three agents may also have affected reporting patterns, particularly given the more recent introduction of tirzepatide.

An additional limitation relates to time-on-market asymmetry and stimulated reporting, commonly described as the Weber effect, whereby newly marketed medicines may experience increased reporting of adverse events during the early post-approval period due to heightened regulatory attention and pharmacovigilance monitoring [10].

In the European Union, liraglutide was approved in 2009, semaglutide in 2018, and tirzepatide in 2022; therefore, tirzepatide has accumulated substantially less post-marketing exposure time than the other agents analyzed. Differences in market presence and cumulative patient exposure may influence reporting intensity across System Organ Classes, particularly for outcomes subject to heightened early monitoring such as hepatobiliary, gastrointestinal, or immune-related events. Consequently, part of the observed disproportionality may reflect differential pharmacovigilance attention rather than intrinsic differences in drug safety profiles [10,25].

Another limitation concerns differences in approved indications and real-world treatment populations across the three agents. Tirzepatide, semaglutide, and liraglutide are used for both type 2 diabetes and chronic weight management, but the relative distribution of these indications may differ across drugs and over time. Indication mix is a recognized confounder in pharmacovigilance disproportionality analyses because reporting patterns may reflect characteristics of the treated population rather than intrinsic drug effects [57,58].

Differences in baseline characteristics such as body mass index, metabolic status, and comorbidity burden may therefore influence reporting in specific System Organ Classes. For example, rapid weight reduction may contribute to musculoskeletal complaints or gallbladder-related events, while psychiatric reports may reflect underlying obesity-related comorbidities rather than direct pharmacological effects. Because indication and clinical context cannot be determined from aggregated spontaneous reporting data, these potential confounders could not be adjusted for in the present analysis.

In addition, analysis at the System Organ Class level limits clinical specificity and may mask heterogeneity at the preferred-term level. A fundamental limitation of spontaneous reporting databases is the absence of exposure denominators, meaning that the number of patients treated with each drug is unknown. Consequently, disproportionality measures such as reporting odds ratios reflect reporting patterns rather than incidence or comparative risk, and elevated RORs may arise from differences in reporting propensity or exposure volume. Finally, spontaneous reporting systems lack exposure denominators and detailed clinical covariates, including information on patient comorbidities, treatment duration, and concomitant therapies, which prevented adjustment for potential confounding factors. Accordingly, the findings should be interpreted as hypothesis-generating and require confirmation in well-designed pharmacoepidemiologic and prospective studies.

Despite these constraints, the study has several notable strengths. This analysis represents one of the first large-scale comparative pharmacovigilance evaluations of SOC-level reporting patterns for tirzepatide relative to established GLP-1 receptor agonists using a consistent analytical framework. The use of EudraVigilance enabled assessment of safety signals in a large and heterogeneous real-world population beyond the confines of randomized clinical trials. The pairwise comparative design provided additional context for interpreting reporting differences across pharmacologically related agents, reducing the likelihood of attributing class-related effects to individual drugs. Finally, integration of disproportionality findings with mechanistic considerations related to dual incretin receptor signaling offers a biologically grounded basis for hypothesis generation and future pharmacoepidemiologic and experimental research.

## 4. Materials and Methods

### 4.1. Data Source

This study was conducted using publicly available aggregated data from the European Medicines Agency (EMA) ADRreports portal [59], which provides structured access to EudraVigilance Individual Case Safety Report (ICSR) summaries. EudraVigilance is the European Union pharmacovigilance database that collects spontaneous reports of suspected adverse drug reactions (ADRs) from healthcare professionals and non-healthcare professionals within and outside the European Economic Area (EEA). It represents a large-scale pharmacovigilance system with global coverage and continuously updated reporting.

The ADRreports interface allows substance-based queries and returns aggregated counts of ICSRs stratified by demographic characteristics (age group, sex), reporter type, geographic origin, seriousness, and System Organ Class (SOC) distribution of reported reactions. Each ICSR corresponds to a unique case report that may include one or more suspected ADRs.

For this analysis, all available ICSRs were extracted for tirzepatide, semaglutide, and liraglutide. No filters were applied and all reports available in the database at the time of extraction were included. Data extraction was performed on [25 January 2026], corresponding to the most recent dataset available at that time.

### 4.2. Study Design

This was a retrospective comparative pharmacovigilance study designed to evaluate SOC-level reporting patterns for tirzepatide relative to semaglutide and liraglutide.

The analysis was conducted exclusively at the System Organ Class level because the ADRreports portal provides publicly accessible outputs primarily structured at this hierarchical level, ensuring reproducibility and consistency across substances. The unit of analysis was the ICSR.

Within each SOC, reports were counted once per ICSR based on the presence or absence of at least one reaction mapped to that SOC. Consequently, a single ICSR could contribute to multiple SOC categories, reflecting multi-system reporting, while duplicate counting within the same SOC was inherently avoided by the structure of the aggregated outputs. Pairwise comparisons were performed between tirzepatide and semaglutide, and between tirzepatide and liraglutide.

No reclassification, recoding, or modification of MedDRA terms was undertaken beyond the SOC categorization provided in the ADRreports portal.

This study was conducted and reported in accordance with the READUS-PV guideline for disproportionality analyses based on spontaneous reporting systems [10]. Detailed compliance with these reporting standards is provided in Supplementary Table S2.

### 4.3. Descriptive Analysis

For each substance, ICSRs were summarized according to age group, sex, geographic origin (EEA vs. non-EEA), reporter type (healthcare professional vs. non-healthcare professional), seriousness classification, and SOC distribution, as presented in the aggregated database outputs.

Because an individual ICSR may include ADRs spanning multiple SOCs, the cumulative number of SOC entries may exceed the total number of ICSRs. Reporting complexity was quantified as the mean number of SOCs per ICSR, calculated as the total number of SOC occurrences divided by the total number of ICSRs for each substance.

In addition, the most frequently reported preferred terms within selected SOC were identified and ranked according to their reporting frequency. For each SOC, the five most frequently reported reactions were extracted for descriptive comparison across the three substances. Preferred terms (PTs) were summarized descriptively using line-listing data extracted from EudraVigilance. PT frequencies were calculated relative to the total number of ICSRs for each substance.

### 4.4. Disproportionality Analysis

Comparative disproportionality analyses were performed using the reporting odds ratio (ROR), a standard signal detection measure in spontaneous reporting systems. Semaglutide and liraglutide were selected as clinically relevant comparators within the same therapeutic class (GLP-1 receptor agonists). For each SOC and pairwise drug comparison, two-by-two contingency tables were constructed from the aggregated counts of ICSRs with and without the SOC of interest for each substance. The unit of analysis was the ICSR.

The ROR was calculated as:

ROR = (a/c) ÷ (b/d)(1)

where

a = number of ICSRs for tirzepatide reporting the SOC
b = number of ICSRs for the comparator reporting the SOC
c = number of tirzepatide ICSRs without the SOC
d = number of comparator ICSRs without the SOC

Confidence intervals (95%) were derived using the standard error of the log-transformed ROR. A signal was considered present when the 95% confidence interval did not include 1.0.

To address multiplicity across SOC comparisons, false discovery rate (FDR) correction using the Benjamini–Hochberg procedure was applied separately within each pairwise comparison. Sensitivity analyses were conducted by restricting calculations to serious reports and reports submitted by healthcare professionals. SOC-level signals were considered fully robust when statistical significance persisted after FDR adjustment and under both sensitivity restrictions.

### 4.5. Statistical Software and Analytical Considerations

All calculations were performed using Microsoft Excel and JASP (version 0.19.3). Forest plots were generated using log-transformed ROR values for visualization purposes, with exponential back-transformation applied for presentation in the ROR scale. No pooled meta-analytic estimates were generated.

The analysis was descriptive and exploratory in nature. Given the inherent limitations of spontaneous reporting systems, including under-reporting, reporting bias, lack of exposure denominators, and absence of detailed clinical covariates, results were interpreted as hypothesis-generating rather than as estimates of incidence or causal association.

## 5. Conclusions

In this EudraVigilance-based analysis, tirzepatide demonstrated a distinct and reproducible SOC-level reporting profile compared with semaglutide and liraglutide. Significant disproportionality signals persisted after false discovery rate adjustment and across serious-case and healthcare professional–restricted sensitivity analyses, supporting the robustness of the observed multi-organ reporting differences. Hepatobiliary, immune system, and musculoskeletal disorders showed the most consistent excess reporting, whereas neoplasms demonstrated reduced reporting odds versus both comparators.

These stable cross-stratum patterns suggest that dual GIP/GLP-1 receptor agonism may be associated with a differentiated safety-reporting signature within the incretin class. The findings are consistent with emerging concepts of functional diversification in incretin pharmacology, whereby differences in receptor engagement and downstream signaling may contribute to structured variation in post-marketing signal architecture rather than a uniform class effect. In this context, pharmacovigilance analyses can provide complementary real-world insights into potential organ-system safety patterns that may not be fully captured in randomized clinical trials.

Although spontaneous reporting systems do not allow estimation of incidence or causality, the consistency, biological plausibility, and triangulation of these signals with regulatory and clinical evidence provide a hypothesis-generating framework. Differences in exposure, treatment indications, and reporting dynamics may also contribute to the observed disproportionality patterns and should be considered when interpreting SOC-level safety signals.

Further exposure-adjusted pharmacoepidemiologic and mechanistic studies are warranted to clarify whether the observed reporting heterogeneity reflects true physiological differentiation under dual incretin receptor activation. Future research integrating real-world cohort analyses, exposure-adjusted comparative studies, and mechanistic investigations of incretin receptor signaling may help determine whether dual GIP/GLP-1 receptor agonism produces distinct organ-system safety patterns or whether the observed signals primarily reflect contextual factors related to treatment populations and pharmacovigilance surveillance.

## Figures and Tables

**Table 1:** Number of individual case safety reports by age group.

Age Group	Tirzepatide n (%)	Semaglutide n (%)	Liraglutide n (%)
Not specified	10,679 (40.3)	16,855 (35.6)	7555 (35.9)
0–1 month	3 (0.0)	8 (0.0)	6 (0.0)
2 months–2 years	4 (0.0)	11 (0.0)	5 (0.0)
3–11 years	3 (0.0)	24 (0.1)	12 (0.1)
12–17 years	18 (0.1)	117 (0.2)	102 (0.5)
18–64 years	12,773 (48.2)	20,697 (43.7)	9811 (46.6)
65–85 years	2914 (11.0)	9334 (19.7)	3479 (16.5)
>85 years	89 (0.3)	291 (0.6)	76 (0.4)
Total	26,483 (100.0)	47,337 (100.0)	21,046 (100.0)

**Table 2:** Number of individual case safety reports by sex.

Sex	Tirzepatide n (%)	Semaglutide n (%)	Liraglutide n (%)
Female	16,744 (63.2)	27,973 (59.1)	12,776 (60.7)
Male	6209 (23.4)	15,762 (33.3)	6818 (32.4)
Not specified	3530 (13.3)	3602 (7.6)	1452 (6.9)
Total	26,483 (100.0)	47,337 (100.0)	21,046 (100.0)

**Table 3:** Geographic origin of reports (EEA vs. non-EEA).

Geographic Origin	Tirzepatide n (%)	Semaglutide n (%)	Liraglutide n (%)
European Economic Area	8324 (31.4)	20,336 (43.0)	8503 (40.4)
Non-EEA	18,159 (68.6)	27,001 (57.0)	12,542 (59.6)
Not specified	0 (0.0)	0 (0.0)	1 (0.0)
Total	26,483 (100.0)	47,337 (100.0)	21,046 (100.0)

**Table 4:** Distribution of adverse event reports by reporter type across tirzepatide, semaglutide, and liraglutide.

Reporter Group	Tirzepatide n (%)	Semaglutide n (%)	Liraglutide n (%)
Healthcare professional	16,221 (61.3)	24,364 (51.5)	13,480 (64.1)
Non-healthcare professional	10,262 (38.7)	22,973 (48.5)	7565 (35.9)
Not specified	0 (0.0)	0 (0.0)	1 (0.0)
Total	26,483 (100.0)	47,337 (100.0)	21,046 (100.0)

**Table 5:** Overall seriousness of reports and serious-to-non-serious ratio.

Measure	Tirzepatide	Semaglutide	Liraglutide
Serious reports, n (%)	19,456 (73.5)	31,417 (66.4)	15,459 (73.5)
Non-serious reports, n (%)	7027 (26.5)	15,920 (33.6)	5584 (26.5)
Serious/Non-serious ratio	2.77	1.97	2.77

**Table 6:** Distribution of individual case safety reports by system organ class for tirzepatide, semaglutide, and liraglutide.

System Organ Class	Tirzepatide n (%)	Semaglutide n (%)	Liraglutide n (%)	Total n (%)
Gastrointestinal disorders	12,583 (47.5)	24,298 (51.3)	9735 (46.3)	46,616 (49.1)
General disorders and administration site conditions	5456 (20.6)	9049 (19.1)	4223 (20.1)	18,728 (19.7)
Injury, poisoning and procedural complications	3779 (14.3)	11,596 (24.5)	2493 (11.9)	17,868 (18.8)
Metabolism and nutrition disorders	3153 (11.9)	6989 (14.8)	2635 (12.5)	12,777 (13.5)
Investigations	3171 (12.0)	6639 (14.0)	2957 (14.1)	12,767 (13.5)
Nervous system disorders	3354 (12.7)	6450 (13.6)	2354 (11.2)	12,158 (12.8)
Psychiatric disorders	2401 (9.1)	4583 (9.7)	1159 (5.5)	8143 (8.6)
Skin and subcutaneous tissue disorders	1794 (6.8)	2915 (6.2)	1243 (5.9)	5952 (6.3)
Hepatobiliary disorders	1970 (7.4)	2123 (4.5)	1219 (5.8)	5312 (5.6)
Musculoskeletal and connective tissue disorders	1521 (5.7)	2256 (4.8)	763 (3.6)	4540 (4.8)
Neoplasms benign, malignant and unspecified	681 (2.6)	1474 (3.1)	2232 (10.6)	4387 (4.6)
Renal and urinary disorders	1114 (4.2)	1877 (4.0)	1056 (5.0)	4047 (4.3)
Infections and infestations	1372 (5.2)	2336 (4.9)	1066 (5.1)	4774 (5.0)
Cardiac disorders	1084 (4.1)	1905 (4.0)	948 (4.5)	3937 (4.1)
Respiratory, thoracic and mediastinal disorders	816 (3.1)	1590 (3.4)	668 (3.2)	3074 (3.2)
Surgical and medical procedures	25 (0.1)	2256 (4.8)	747 (3.6)	3028 (3.2)
Vascular disorders	852 (3.2)	1342 (2.8)	550 (2.6)	2744 (2.9)
Reproductive system and breast disorders	564 (2.1)	698 (1.5)	236 (1.1)	1498 (1.6)
Immune system disorders	615 (2.3)	565 (1.2)	340 (1.6)	1520 (1.6)
Product issues	103 (0.4)	1116 (2.4)	412 (2.0)	1631 (1.7)
Eye disorders	1025 (3.9)	3182 (6.7)	585 (2.8)	4792 (5.1)
Endocrine disorders	174 (0.7)	346 (0.7)	270 (1.3)	790 (0.8)
Ear and labyrinth disorders	192 (0.7)	415 (0.9)	136 (0.6)	743 (0.8)
Blood and lymphatic system disorders	256 (1.0)	328 (0.7)	224 (1.1)	808 (0.9)
Pregnancy, puerperium and perinatal conditions	146 (0.6)	215 (0.5)	119 (0.6)	480 (0.5)
Congenital, familial and genetic disorders	38 (0.1)	96 (0.2)	65 (0.3)	199 (0.2)
Social circumstances	22 (0.1)	582 (1.2)	146 (0.7)	750 (0.8)

Percentages are calculated based on the total number of ICSRs for each substance.

**Table 7:** Reporting complexity expressed as number of System Organ Classes per individual case safety report.

Measure	Tirzepatide	Semaglutide	Liraglutide	Overall
Total ICSRs, n	26,483	47,337	21,046	94,866
Total SOC occurrences, n	48,261	97,221	38,581	184,063
Mean SOCs per ICSR	1.82	2.05	1.83	1.94

**Table 8:** Pairwise reporting odds ratios (RORs) by System Organ Class (SOC).

System Organ Class	ROR Tirzepatide vs. Semaglutide (95% CI)	ROR Tirzepatide vs. Liraglutide (95% CI)
Blood and lymphatic system disorders	1.40 (1.19–1.65)	1.13 (0.94–1.35)
Cardiac disorders	1.02 (0.94–1.10)	1.13 (1.03–1.23)
Congenital, familial and genetic disorders	0.71 (0.49–1.03)	0.57 (0.38–0.86)
Ear and labyrinth disorders	0.83 (0.70–0.98)	1.39 (1.12–1.73)
Endocrine disorders	0.90 (0.75–1.08)	0.63 (0.52–0.76)
Eye disorders	0.56 (0.52–0.60)	1.75 (1.58–1.94)
Gastrointestinal disorders	0.86 (0.83–0.88)	1.52 (1.46–1.57)
General disorders and administration site conditions	1.10 (1.06–1.14)	1.34 (1.28–1.40)
Hepatobiliary disorders	1.71 (1.61–1.82)	1.64 (1.52–1.76)
Immune system disorders	1.97 (1.75–2.21)	1.80 (1.57–2.05)
Infections and infestations	1.05 (0.98–1.13)	1.28 (1.18–1.39)
Injury, poisoning and procedural complications	0.51 (0.49–0.53)	1.57 (1.49–1.66)
Investigations	0.83 (0.80–0.87)	1.06 (1.01–1.12)
Metabolism and nutrition disorders	0.78 (0.75–0.82)	1.20 (1.14–1.27)
Musculoskeletal and connective tissue disorders	1.22 (1.14–1.30)	2.02 (1.85–2.21)
Neoplasms that are benign, malignant and unspecified (incl. cysts and polyps)	0.82 (0.75–0.90)	0.28 (0.26–0.31)
Nervous system disorders	0.92 (0.88–0.96)	1.46 (1.38–1.54)
Pregnancy, puerperium and perinatal conditions	1.21 (0.98–1.50)	1.21 (0.95–1.54)
Product issues	0.16 (0.13–0.20)	0.24 (0.20–0.30)
Psychiatric disorders	0.93 (0.88–0.98)	2.14 (1.99–2.30)
Renal and urinary disorders	1.06 (0.99–1.15)	1.04 (0.95–1.13)
Reproductive system and breast disorders	1.45 (1.30–1.63)	2.38 (2.04–2.77)
Respiratory, thoracic and mediastinal disorders	0.91 (0.84–1.00)	1.21 (1.09–1.34)
Skin and subcutaneous tissue disorders	1.11 (1.04–1.18)	1.45 (1.35–1.56)
Social circumstances	0.07 (0.04–0.10)	0.15 (0.09–0.23)
Surgical and medical procedures	0.02 (0.01–0.03)	0.03 (0.02–0.05)
Vascular disorders	1.14 (1.04–1.24)	1.54 (1.38–1.72)

Reporting odds ratios (RORs) were calculated using a case/non-case approach within the EudraVigilance database. Disproportionality signals were considered present when the 95% confidence interval did not include unity. RORs reflect reporting patterns and should not be interpreted as incidence or comparative clinical risk.

**Table 9:** Intersection of fully robust system organ class–level disproportionality signals across pairwise comparisons.

System Organ Class	TIR vs. SEMA Total ROR (95% CI)	Serious ROR (95% CI)	HCP ROR (95% CI)	TIR vs. LIRA Total ROR (95% CI)	Serious ROR (95% CI)	HCP ROR (95% CI)	Directionality Pattern
Eye disorders	0.56	0.51	0.51	1.75	1.40	2.12	Comparator-dependent
	(0.52–0.60)	(0.47–0.56)	(0.46–0.56)	(1.58–1.94)	(1.25–1.56)	(1.81–2.48)	
Gastrointestinal disorders	0.86	0.96	0.88	1.52	1.10	0.83	Comparator-dependent
	(0.83–0.88)	(0.93–1.00)	(0.84–0.91)	(1.46–1.57)	(1.05–1.15)	(0.79–0.87)	
Hepatobiliary disorders	1.71	1.62	1.71	1.64	1.36	1.30	Consistently elevated
	(1.61–1.82)	(1.52–1.73)	(1.58–1.85)	(1.52–1.76)	(1.26–1.47)	(1.19–1.42)	
Immune system disorders	1.97	1.80	1.77	1.80	1.50	1.28	Consistently elevated
	(1.75–2.21)	(1.59–2.04)	(1.51–2.06)	(1.57–2.05)	(1.30–1.74)	(1.08–1.52)	
Injury, poisoning and procedural complications	0.51	0.46	0.52	1.57	1.16	1.16	Comparator-dependent
	(0.49–0.53)	(0.44–0.49)	(0.49–0.55)	(1.49–1.66)	(1.09–1.24)	(1.07–1.26)	
Metabolism and nutrition disorders	0.78	0.86	0.80	1.20	1.13	0.86	Comparator-dependent
	(0.75–0.82)	(0.82–0.90)	(0.75–0.85)	(1.14–1.27)	(1.06–1.20)	(0.80–0.92)	
Musculoskeletal and connective tissue disorders	1.22	1.18	1.17	2.02	1.51	1.58	Consistently elevated
	(1.14–1.30)	(1.09–1.28)	(1.06–1.30)	(1.85–2.21)	(1.37–1.67)	(1.39–1.79)	
Neoplasms benign, malignant and unspecified (incl. cysts and polyps)	0.82	0.73	0.64	0.28	0.21	0.21	Consistently reduced
	(0.75–0.90)	(0.67–0.80)	(0.57–0.73)	(0.26–0.31)	(0.19–0.23)	(0.19–0.24)	

Fully robust signals were defined as SOC-level associations remaining statistically significant after false discovery rate (Benjamini–Hochberg) correction in the primary analysis and persisting under both serious-case–restricted and healthcare-professional–restricted conditions. RORs reflect reporting disproportionality within the EudraVigilance database and should not be interpreted as incidence or comparative clinical risk.

**Table 10:** Leading preferred terms within the eight fully robust SOCs.

Preferred Term (PT)	Tirzepatide n (%) (N = 26,483)	Semaglutide n (%) (N = 47,337)	Liraglutide n (%) (N = 21,046)
Eye disorders			
Blindness	102 (0.39%)	270 (0.57%)	46 (0.22%)
Vision blurred	59 (0.22%)	–	–
Visual impairment	51 (0.19%)	121 (0.26%)	13 (0.06%)
Blindness unilateral	46 (0.17%)	–	–
Optic ischaemic neuropathy	46 (0.17%)	339 (0.72%)	17 (0.08%)
Cataract	–	172 (0.36%)	69 (0.33%)
Diabetic retinopathy	–	101 (0.21%)	16 (0.08%)
Gastrointestinal disorders			
Abdominal pain	1449 (5.47%)	2248 (4.75%)	651 (3.09%)
Diarrhoea	1175 (4.44%)	1814 (3.83%)	540 (2.57%)
Pancreatitis	1082 (4.09%)	1413 (2.99%)	–
Pancreatitis acute	–	–	554 (2.63%)
Abdominal pain upper	878 (3.32%)	1772 (3.74%)	–
Constipation	635 (2.40%)	–	–
Nausea	–	1480 (3.13%)	461 (2.19%)
Hepatobiliary disorders			
Cholelithiasis	747 (2.82%)	521 (1.10%)	329 (1.56%)
Cholecystitis	235 (0.89%)	229 (0.48%)	100 (0.48%)
Biliary colic	82 (0.31%)	107 (0.23%)	38 (0.18%)
Cholecystitis acute	76 (0.29%)	71 (0.15%)	40 (0.19%)
Gallbladder disorder	67 (0.25%)	63 (0.13%)	–
Drug-induced liver injury	–	–	34 (0.16%)
Immune system disorders			
Anaphylactic reaction	166 (0.63%)	94 (0.20%)	53 (0.25%)
Hypersensitivity	147 (0.55%)	120 (0.25%)	76 (0.36%)
Drug hypersensitivity	59 (0.22%)	41 (0.09%)	33 (0.16%)
Anaphylactic shock	58 (0.22%)	34 (0.07%)	24 (0.11%)
Autoimmune disorder	12 (0.05%)	22 (0.05%)	–
Amyloidosis	–	–	5 (0.02%)
Injury, poisoning and procedural complications			
Incorrect dose administered	158 (0.60%)	–	–
Off-label use	148 (0.56%)	398 (0.84%)	89 (0.42%)
Contusion	64 (0.24%)	–	–
Intentional product misuse	51 (0.19%)	–	–
Fall	48 (0.18%)	120 (0.25%)	45 (0.21%)
Inappropriate schedule of product administration	–	229 (0.48%)	46 (0.22%)
Product use in unapproved indication	–	117 (0.25%)	–
Accidental overdose	–	106 (0.22%)	24 (0.11%)
Drug dose titration not performed	–	–	23 (0.11%)
Metabolism and nutrition disorders			
Dehydration	426 (1.61%)	526 (1.11%)	163 (0.77%)
Decreased appetite	270 (1.02%)	926 (1.96%)	186 (0.88%)
Hypoglycaemia	203 (0.77%)	261 (0.55%)	131 (0.62%)
Diabetic ketoacidosis	106 (0.40%)	229 (0.48%)	109 (0.52%)
Ketoacidosis	57 (0.22%)	–	–
Diabetes mellitus inadequate control	–	128 (0.27%)	125 (0.59%)
Musculoskeletal and connective tissue disorders			
Arthralgia	205 (0.77%)	287 (0.61%)	63 (0.30%)
Myalgia	137 (0.52%)	61 (0.13%)	–
Back pain	100 (0.38%)	145 (0.31%)	64 (0.30%)
Muscle spasms	31 (0.12%)	–	10 (0.05%)
Pain in extremity	21 (0.08%)	–	–
Arthritis	–	62 (0.13%)	14 (0.07%)
Arthropathy	–	41 (0.09%)	15 (0.07%)
Neoplasms benign, malignant and unspecified			
Pancreatic neoplasm malignant	45 (0.17%)	62 (0.13%)	35 (0.17%)
Pancreatic carcinoma	41 (0.15%)	73 (0.15%)	38 (0.18%)
Thyroid neoplasm	37 (0.14%)	55 (0.12%)	28 (0.13%)
Pancreatic neoplasm	29 (0.11%)	43 (0.09%)	24 (0.11%)
Benign neoplasm	18 (0.07%)	31 (0.07%)	17 (0.08%)

PT frequencies are presented as number of reports and percentage of total ICSRs for each substance. Because individual ICSRs may contain multiple preferred terms, counts represent PT occurrences rather than unique cases. N is the total number of reports for each drug. n is the number of reports for each preferred term.

**Table 11:** Cross-source evaluation of key SOC-level adverse event signals identified in disproportionality analysis.

System Organ Class	Disproportionality Pattern	Listed in SmPC	Evidence from Published Studies	Overall Interpretation
Hepatobiliary disorders	Increased reporting vs. semaglutide and liraglutide	Cholelithiasis; gallbladder disease	Increased risk of biliary events reported with GLP-1 receptor agonists and rapid weight reduction	Consistent with known incretin-related biliary effects and weight-loss–associated gallbladder risk
Immune system disorders	Increased reporting vs. both comparators	Hypersensitivity reactions; injection-site reactions	Anti-drug antibodies and injection-site reactions described in clinical trials	Likely reflects immunogenicity and hypersensitivity monitoring
Musculoskeletal and connective tissue disorders	Increased reporting vs. both comparators	Arthralgia; musculoskeletal pain	Weight-loss–associated biomechanical and musculoskeletal changes reported	May represent indirect effects of rapid weight reduction
Eye disorders	Lower reporting vs. semaglutide; higher vs. liraglutide	Diabetic retinopathy complications (semaglutide)	Retinopathy progression associated with rapid glycemic improvement	Likely reflects metabolic control dynamics rather than direct ocular toxicity
Gastrointestinal disorders	Lower reporting vs. semaglutide; higher vs. liraglutide	Nausea, vomiting, diarrhea, abdominal pain	Consistently reported across randomized trials and real-world studies	Established pharmacologic class effect
Injury, poisoning and procedural complications	Lower reporting vs. semaglutide; higher vs. liraglutide	Not prominently highlighted	Limited direct corroboration in literature	May reflect treatment context or reporting patterns
Neoplasms benign, malignant and unspecified	Reduced reporting vs. both comparators (markedly vs. liraglutide)	Thyroid C-cell tumor monitoring (liraglutide)	Preclinical rodent findings; uncertain human relevance in humans	Likely reflects historical regulatory surveillance rather than novel signal
Metabolism and nutrition disorders	Lower reporting vs. semaglutide; moderately increased vs. liraglutide	Decreased appetite; hypoglycemia (with insulin/sulfonylureas)	Weight loss and appetite suppression widely documented	Expected pharmacodynamic effect of incretin-based therapies

## Data Availability

The original contributions presented in the study are included in the article; further inquiries can be directed to the corresponding authors.

## Supplementary Materials

The following supporting information can be downloaded at: https://www.mdpi.com/article/10.3390/ijms27072988/s1.

## Abbreviations

The following abbreviations are used in this manuscript:

ADR	Adverse drug reaction
AE	Adverse event
Aib	α-aminoisobutyric acid
AMPK	Amp-activated protein kinase
β-arrestin	Beta-arrestin
CI	Confidence interval
CNS	Central nervous system
CYP7A1	Cholesterol 7 alpha-hydroxylase
EMA	European medicines agency
EV	Eudravigilance
FGF19	Fibroblast growth factor 19
FXR	Harnesoid x receptor
GIP	Glucose-dependent insulinotropic polypeptide
GIPR	Glucose-dependent insulinotropic polypeptide receptor
GLP-1	Glucagon-like peptide-1
GLP-1 RA	Glucagon-like peptide-1 receptor agonist
GLP-1R	Glucagon-like peptide-1 receptor
GPCR	G protein-coupled receptor
ICSR	Individual case safety report
IJMS	International journal of molecular sciences
MedDRA	Medical dictionary for regulatory activities
PK/PD	Pharmacokinetics/pharmacodynamics
PTs	Preferred terms
ROR	Reporting odds ratio
SmPC	Summary of Product Characteristics
SOC	System organ class
SURMOUNT	Tirzepatide clinical trial program for obesity
SURPASS	Tirzepatide clinical trial program for type 2 diabetes
T2DM	Type 2 diabetes mellitus
