# Supplementary material for: Disproportionality Analysis of Tirzepatide vs. Semaglutide and Liraglutide: System Organ Class-Level Post-Marketing Reporting Patterns in EudraVigilance
Source: Int J Mol Sci. 2026 Mar 25;27(7):2988. doi: 10.3390/ijms27072988 (PMC13073578; doi:10.3390/ijms27072988)
Supplement: Supplementary file 1 [file ijms-27-02988-s001.zip › ijms-4200159-supplementary.pdf]

Table S1. Reporting odds ratios (RORs) with 95% confidence intervals for all System Organ Classes (SOCs) in total reports, serious reports, and healthcare professional (HCP) reports for tirzepatide versus semaglutide and tirzepatide versus liraglutide.

SOC	TIR vs SEMA Total	TIR vs LIRA Total	TIR vs SEMA Serious	TIR vs LIRA Serious	TIR vs SEMA HCP	TIR vs LIRA HCP
Blood and lymphatic system disorders	1.40 (1.19–1.65)	1.13 (0.94–1.35)	1.26 (1.06–1.50)	0.87 (0.72–1.05)	1.19 (0.97–1.47)	0.77 (0.62–0.96)
Cardiac disorders	1.02 (0.94–1.10)	1.13 (1.03–1.23)	0.95 (0.87–1.03)	0.91 (0.83–1.00)	1.08 (0.97–1.19)	0.91 (0.81–1.02)
Congenital, familial and genetic disorders	0.71 (0.49–1.03)	0.57 (0.38–0.86)	0.60 (0.41–0.89)	0.43 (0.28–0.64)	0.69 (0.43–1.12)	0.46 (0.28–0.76)
Ear and labyrinth disorders	0.83 (0.70–0.98)	1.39 (1.12–1.73)	0.93 (0.76–1.14)	1.25 (0.96–1.62)	0.68 (0.52–0.88)	1.04 (0.75–1.44)
Endocrine disorders	0.90 (0.75–1.08)	0.63 (0.52–0.76)	0.77 (0.63–0.94)	0.47 (0.38–0.57)	1.02 (0.80–1.30)	0.51 (0.41–0.65)
Eye disorders	0.56 (0.52–0.60)	1.75 (1.58–1.94)	0.51 (0.47–0.56)	1.40 (1.25–1.56)	0.51 (0.46–0.56)	2.12 (1.81–2.48)
Gastrointestinal disorders	0.86 (0.83–0.88)	1.52 (1.46–1.57)	0.96 (0.93–1.00)	1.10 (1.05–1.15)	0.88 (0.84–0.91)	0.83 (0.79–0.87)
General disorders and administration site conditions	1.10 (1.06–1.14)	1.34 (1.28–1.40)	0.89 (0.84–0.95)	1.15 (1.09–1.21)	0.95 (0.89–1.02)	1.02 (0.95–1.09)
Hepatobiliary disorders	1.71 (1.61–1.82)	1.64 (1.52–1.76)	1.62 (1.52–1.73)	1.36 (1.26–1.47)	1.71 (1.58–1.85)	1.30 (1.19–1.42)
Immune system disorders	1.97 (1.75–2.21)	1.80 (1.57–2.05)	1.80 (1.59–2.04)	1.50 (1.30–1.74)	1.77 (1.51–2.06)	1.28 (1.08–1.52)
Infections and infestations	1.05 (0.98–1.13)	1.28 (1.18–1.39)	0.82 (0.76–0.89)	0.99 (0.92–1.07)	0.81 (0.74–0.89)	0.92 (0.84–1.01)
Injury, poisoning and procedural complications	0.51 (0.49–0.53)	1.57 (1.49–1.66)	0.46 (0.44–0.49)	1.16 (1.09–1.24)	0.52 (0.49–0.55)	1.16 (1.07–1.26)
Investigations	0.83 (0.80–0.87)	1.06 (1.01–1.12)	0.87 (0.81–0.94)	1.03 (0.96–1.11)	0.89 (0.81–0.98)	0.97 (0.88–1.06)

Metabolism and nutrition disorders	0.78 (0.75–0.82)	1.20 (1.14–1.27)	0.86 (0.82–0.90)	1.13 (1.06–1.20)	0.80 (0.75–0.85)	0.86 (0.80–0.92)
Musculoskeletal and connective tissue disorders	1.22 (1.14–1.30)	2.02 (1.85–2.21)	1.18 (1.09–1.28)	1.51 (1.37–1.67)	1.17 (1.06–1.30)	1.58 (1.39–1.79)
Neoplasms benign, malignant and unspecified (incl cysts and polyps)	0.82 (0.75–0.90)	0.28 (0.26–0.31)	0.73 (0.67–0.80)	0.21 (0.19–0.23)	0.64 (0.57–0.73)	0.21 (0.19–0.24)
Nervous system disorders	0.92 (0.88–0.96)	1.46 (1.38–1.54)	1.02 (0.97–1.07)	1.28 (1.20–1.36)	0.87 (0.82–0.93)	1.19 (1.10–1.29)
Pregnancy, puerperium and perinatal conditions	1.21 (0.98–1.50)	1.21 (0.95–1.54)	0.70 (0.53–0.92)	0.54 (0.40–0.72)	0.78 (0.55–1.10)	0.59 (0.40–0.87)
Product issues	0.16 (0.13–0.20)	0.24 (0.20–0.30)	0.58 (0.49–0.69)	1.19 (0.99–1.43)	0.60 (0.49–0.73)	1.16 (0.92–1.45)
Psychiatric disorders	0.93 (0.88–0.98)	2.14 (1.99–2.30)	0.90 (0.85–0.95)	1.97 (1.82–2.14)	1.31 (1.22–1.42)	2.14 (1.93–2.37)
Renal and urinary disorders	1.06 (0.99–1.15)	1.04 (0.95–1.13)	0.91 (0.82–1.01)	1.04 (0.92–1.17)	0.93 (0.82–1.06)	1.01 (0.88–1.17)
Reproductive system and breast disorders	1.45 (1.30–1.63)	2.38 (2.04–2.77)	1.71 (1.48–1.97)	2.10 (1.74–2.54)	1.34 (1.14–1.57)	1.79 (1.46–2.19)
Respiratory, thoracic and mediastinal disorders	0.91 (0.84–1.00)	1.21 (1.09–1.34)	0.90 (0.82–0.99)	0.98 (0.88–1.10)	0.96 (0.86–1.08)	0.97 (0.85–1.11)
Skin and subcutaneous tissue disorders	1.11 (1.04–1.18)	1.45 (1.35–1.56)	1.28 (1.18–1.40)	1.15 (1.04–1.27)	1.01 (0.93–1.09)	1.04 (0.95–1.14)
Social circumstances	0.07 (0.04–0.10)	0.15 (0.09–0.23)	0.70 (0.52–0.95)	1.20 (0.90–1.60)	0.81 (0.55–1.19)	1.07 (0.72–1.60)
Surgical and medical procedures	0.02 (0.01–0.03)	0.03 (0.02–0.05)	0.65 (0.54–0.79)	1.09 (0.90–1.32)	0.71 (0.56–0.90)	1.04 (0.80–1.35)
Vascular disorders	1.14 (1.04–1.24)	1.54 (1.38–1.72)	1.12 (1.02–1.23)	1.24 (1.11–1.40)	1.19 (1.06–1.34)	1.27 (1.10–1.46)

ROR, reporting odds ratio; CI, confidence interval; HCP, healthcare professional; TIR, tirzepatide; TIR, tirzepatide; LIRA, liraglutide. Disproportionality signals were considered present when the 95% confidence interval did not include 1.00. Serious reports were defined according to EudraVigilance criteria.

Table S2. Compliance with READUS-PV reporting recommendations for disproportionality analyses.

READUS-PV Domain	Implementation in this study	Location in manuscript
Title	Study identified as a disproportionality analysis using EudraVigilance data; drugs under study clearly specified (tirzepatide, semaglutide, liraglutide)	Title
Background and rationale	Pharmacological context, safety profile, and knowledge gap described; rationale for disproportionality approach and suitability of ICSR database justified	Introduction
Objectives	Objective defined as comparative SOC-level disproportionality analysis of tirzepatide versus GLP-1 receptor agonists	Introduction (final paragraph)
Study design	Retrospective disproportionality analysis using aggregated ICSRs; pairwise comparisons and sensitivity analyses specified	Methods 4.2
Data source	EudraVigilance (EMA ADRreports) described, including public access, global coverage, and MedDRA-based SOC classification	Methods 4.1
Data extraction and preprocessing	Extraction date reported; no filtering or transformation applied; aggregated data used as provided	Methods 4.1
Study population	All available ICSRs included without restriction; sensitivity analyses based on seriousness and reporter type	Methods 4.1, 4.4
Variables	Key variables defined (age, sex, reporter type, seriousness, SOC classification);<br>missing values retained as reported	Methods 4.3
Event and drug classification	Events grouped at SOC level using MedDRA hierarchy; no recoding or reclassification performed	Methods 4.2
Additional data sources	Not applicable (no external data sources used)	—
Descriptive analysis	Demographic and reporting characteristics summarized; SOC distribution and PT frequencies described	Methods 4.3
Disproportionality analysis	ROR calculated using 2×2 contingency tables; ICSRs as unit; signals defined as 95% CI excluding 1.0; FDR correction applied; comparators selected within same therapeutic class	Methods 4.4
Sensitivity analyses	Analyses restricted to serious ICSRs and healthcare professional reports to assess robustness	Methods 4.4
Case-level analysis	Not applicable (aggregated database analysis)	—
Results – descriptive data	Number and characteristics of reports summarized descriptively across drugs	Results (Section 2.1-2.5)
Results – disproportionality	ROR estimates with 95% CI and FDR-adjusted results presented; sensitivity analyses reported	Results (Section 2.6 - 2.6.1-2.6.2)
Interpretation of findings	SOC-level signals interpreted, distinguishing expected effects from emerging signals and contextualized with literature	Discussion

External validity and implications	Generalizability, clinical relevance, and need for further studies discussed	Discussion
Limitations	Limitations of spontaneous reporting systems and disproportionality analyses explicitly addressed	Discussion (Limitations)
